# Leveraging Available Resources and Stakeholder Involvement for Improved Productivity of African Livestock in the Era of Genomic Breeding

**DOI:** 10.3389/fgene.2019.00357

**Published:** 2019-04-24

**Authors:** Eveline M. Ibeagha-Awemu, Sunday O. Peters, Martha N. Bemji, Matthew A. Adeleke, Duy N. Do

**Affiliations:** ^1^Sherbrooke Research and Development Centre, Agriculture and Agri-Food Canada, Sherbrooke, QC, Canada; ^2^Department of Animal Science, Berry College, Mount Berry, GA, United States; ^3^Department of Animal Breeding and Genetics, Federal University of Agriculture, Abeokuta, Abeokuta, Nigeria; ^4^School of Life Sciences, University of Kwazulu-Natal, Durban, South Africa

**Keywords:** Africa, breed improvement, genomic breeding, stakeholders, sustainable livestock development

## Abstract

The African continent is home to diverse populations of livestock breeds adapted to harsh environmental conditions with more than 70% under traditional systems of management. Animal productivity is less than optimal in most cases and is faced with numerous challenges including limited access to adequate nutrition and disease management, poor institutional capacities and lack of adequate government policies and funding to develop the livestock sector. Africa is home to about 1.3 billion people and with increasing demand for animal proteins by an ever growing human population, the current state of livestock productivity creates a significant yield gap for animal products. Although a greater section of the population, especially those living in rural areas depend largely on livestock for their livelihoods; the potential of the sector remains underutilized and therefore unable to contribute significantly to economic development and social wellbeing of the people. With current advances in livestock management practices, breeding technologies and health management, and with inclusion of all stakeholders, African livestock populations can be sustainably developed to close the animal protein gap that exists in the continent. In particular, advances in gene technologies, and application of genomic breeding in many Western countries has resulted in tremendous gains in traits like milk production with the potential that, implementation of genomic selection and other improved practices (nutrition, healthcare, etc.) can lead to rapid improvement in traits of economic importance in African livestock populations. The African livestock populations in the context of this review are limited to cattle, goat, pig, poultry, and sheep, which are mainly exploited for meat, milk, and eggs. This review examines the current state of livestock productivity in Africa, the main challenges faced by the sector, the role of various stakeholders and discusses in-depth strategies that can enable the application of genomic technologies for rapid improvement of livestock traits of economic importance.

## Introduction

The African continent is home to diverse populations of livestock breeds adapted to their local environments in diverse agro-ecological zones. The diversity of the various cattle, sheep, goat, pig, and chicken breeds since their introduction or domestication has been shaped by a delicate balance between human and natural selection, and environmental adaptation. Livestock are central to the Africa society and economy and serve diverse roles such as: (1) source of food (provides meat and milk in the diet); (2) income generation through sale of meat, milk, and hide; (3) savings and insurance; (4) source of draft power and manure in crop production; (5) a means of transportation; (6) use in festivals and traditional ceremonies (marriage, birth, death, coronation, and initiation ceremonies) and, (7) source of power, pride, prestige, and status. Despite these benefits, livestock productivity is less than optimal, not sustainable and unable to match demand and population growth.

**FIGURE 1:**
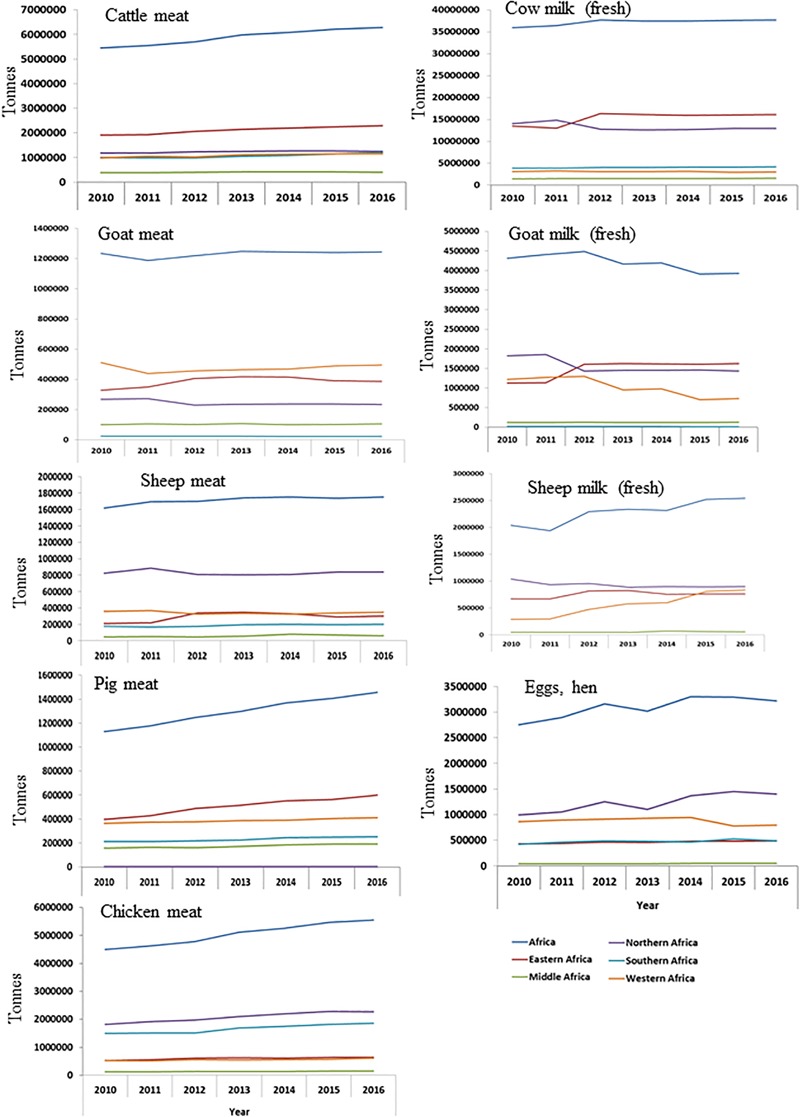
Trends in livestock productivity (meat and milk) in Africa and regions from 2010 to 2016.

The Food and Agricultural Organization (FAO) estimates the total African population at 1.3 billion in 2017 (FAOSTATS, 2018) with rural and urban populations of 717 million and 505 million, respectively. Furthermore, the urban population has witnessed a steady annual increase of 3.59% since 2010, as compared to 1.74% annual rural population increase, and these increases are accompanied by increased demand for animal products. To meet this demand in the face of low productivity of livestock, African governments have increased imports of cattle meat from 482,111 tons in 2012 to 612,353 tons in 2016 and pig meat from 184,322 tons in 2012 to 252,611 tons in 2016 (Table 1). The populations of cattle, sheep, goat, pig, and chicken in the African continent and the various regions in 2016 are shown in Table 2. Similarly, the statistics on livestock productivity (meat, milk, and eggs) from 2010 are shown in Figure 1. To position the livestock sector to adequately contribute to food supply and economic development of the continent, measures must be taken to ensure sustainability in African livestock production systems which form part of FAO’s strategic objectives^1^. The livestock sector has the potential to enhance the livelihoods of Africa’s rural poor and genomic selection can play a key role. Given that the human population growth of the continent is higher than its food protein production, the need for targeted action to increase livestock productivity has never been greater, and genomic selection may play a significant role.

**Table 1:** Statistics^∗^ on major meat and livestock products imported by Africa in the period 2012–2016.

		Bovine	Cheese	Eggs in	Pig meat	Poultry meat	Sheep meat	Ovine meat	Milk
Year	Unit	meat	and curd	the shell	(fresh)	(fresh)	(fresh)	(fresh)	equivalent
2012	Tons	482,111	128783	91,367	184,322	NA	29,326	29,326	8,683,819
2013	Tons	549,127	159867	70,445	217,845	1,646,964	34,713	34,713	8,179,410
2014	Tons	866,902	155154	86,134	775,640	2,057,367	35,095	35,095	10,006,915
2015	Tons	700,110	160501	77,701	483,221	1,725,336	32,259	32,259	9,915,196
2016	Tons	612,353	148419	63,096	252,611	1,680,672	28,112	28,112	9,437,991

*^∗^FAOSTATS, 2018, http://www.fao.org/faostat/.*

**Table 2:** Livestock population^∗^ in Africa and regions in year 2016.

Species	Africa		Eastern Africa		Middle Africa		Northern Africa		Southern Africa		Western Africa	
		Annual		Annual		Annual		Annual		Annual		
	Total	increase^∗∗^	Total	increase	Total	increase	Total	increase	Total	increase	Total	Changes
Cattle	324,844,768	2.14%	165,472,085	4.37%	24,633,591	1.72%	41,755,788	-3.76%	19,298,301	-3.76%	73,685,002	2.95%
Chicken	1,903,550,000	2.69%	3,64,295,000	3.26%	128,523,000	4.21%	660,049,000	2.98%	183,689,000	2.98%	566,994,000	3.80%
Goat	387,667,193	2.71%	142,956,328	4.25%	27,771,055	0.63%	49,987,818	0.25%	9,848,516	0.25%	157,103,476	3.04%
Pig	36,625,241	3.30%	13,895,837	5.56%	7,584,063	2.26%	28,169	-0.88%	1,700,072	-0.88%	13,417,100	2.54%
Sheep	351,579,045	2.06%	92,885,970	6.69%	13,307,827	6.93%	107,971,977	-1.12%	27,074,442	-1.12%	110,338,829	2.94%

*^∗^FAOSTATS, 2018, http://www.fao.org/faostat/. ^∗∗^Percent average annual increase in livestock populations from 2011 to 2016.*

## African Livestock Productivity in the Era of Genomic Breeding

### State of Genomic Breeding Application in Western Countries

Genomic breeding in simple terms refers to the inclusion of deoxyribonucleic acid (DNA) or genomic information to select superior animals and make them parents of the next generation (Meuwissen et al., 2001, 2016). Thus, genomic selection simply is application of the knowledge of genetic variations found in the genome and their relationship with traits or phenotypes (e.g., milk yield, body weight, egg size, etc.) in selection for improved productivity (e.g., litter size, milk yield, etc.). The application of quantitative genetic theories, statistical approaches, artificial insemination and organized breeding practices resulted to rapid gains in livestock traits in the last nine decades (Blasco, 2013; Hill, 2014; Oldenbroek and Waaij, 2014; Weller, 2016). Mostly, the exact mechanisms behind these gains were not known but with the discovery of the DNA structure and developments in DNA sequencing and genotyping techniques, knowledge on the association between DNA variations and livestock traits began to emerge. Thus, with increasing demands for animal products by an ever growing population and changing societal needs, the animal breeding act needed to evolve to incorporate genomic information in order to speed up response and increase productivity.

Genomic breeding started with the application of marker assisted selection considering a few markers at a time and has evolved to the use of thousands of markers and even whole genome data (Hayes and Goddard, 2001; Hayes et al., 2013). Genomic selection entails the estimation of breeding values from markers spanning the entire genome. The estimation of marker effects is carried out within a reference population (a population of individuals with phenotype and marker genotype information). These effects are then applied to select candidates with marker genotype information without phenotypes to estimate genomic breeding value (GEBV). The reliability and accuracy of this approach depends on many factors including the number of individuals genotyped, the density of the markers on the genome, effective population size, the genetic relationship between the reference and predicted populations, the nature of the traits and the applied methods, etc. (Habier et al., 2007, 2011; Bolormaa et al., 2013a,b, 2014; Meuwissen et al., 2016). Genomic selection for milk and beef traits has been successfully implemented in several countries including Australia, Canada, France, Germany, Great Britain, Ireland, the Netherlands, New Zealand, the Scandinavian countries and the United States of America (Silva et al. (2014); Weller et al., 2017). Genomic breeding application in these countries is facilitated by many factors including: (1) large population of animals; (2) specialized farms; (3) comprehensive data on animals; (4) access to genotyping platforms which makes single nucleotide polymorphism (SNP) genotyping more cost effective; (5) available resources (finance, technical knowhow) to accomplish genotyping; (6) existence of breed associations; (7) application of artificial insemination; (8) development of breeds for specific purposes; (9) large scale international breeding companies that sell semen from high performing males for use in breeding for specific traits; (10) creation of farmer organizations, (11) implementation of national evaluation schemes; (12) development of statistical models to handle large data and (13) computing infrastructure to deliver genomics information which helps to facilitate genetic gains in livestock.

In addition, several initiatives have been undertaken to make available data on all sources of genomic variation in livestock genomes to further increase the success of genomic breeding. Such efforts include, but not limited to, the 1,000 bull genome project with aim to re-sequence the whole genomes of 1,000 bulls and has already made available about 84 million SNPs and 2.5 million small insertions/deletions (Hayes and Daetwyler, 2019) and the international consortium for Functional Annotation of Animal Genomes (FAANG^2^) established to provide the infrastructure to detect and proficiently analyze genome wide functional regulatory elements (DNA methylation, histone modifications, chromatin remodeling, non-coding RNA) in animal genomes (cattle, chicken, goat, pig, and sheep) necessary to understand how variation in gene sequences and functional components determines phenotypic diversity, and how this is translated into complex phenotypes; and thus fill the genotype-to-phenotype gap that is missing in current livestock improvement programs (Andersson et al., 2015; Tuggle et al., 2016). Major gains achieved with the use of genomic information and implementation of genomic selection include higher rate of genetic gain, increased reliability of predicting breeding values, higher intensity of selection, shortened generation interval, selection of animals possible at early age, and rapid genetic improvement in lowly heritable traits (e.g., fertility, lifespan, health, etc.) (reviewed by Hayes et al., 2009; Ibeagha-Awemu and Khatib, 2017; Weller et al., 2017; Mrode et al., 2018).

The application of genomic selection in Western countries and the advances that have been made in breeding (e.g., dairy traits) have been driven by the economic needs of the producers. However, challenges regarding sustainability of livestock production necessitate consideration of the economic, societal and environmental factors. A focus on increased milk production for example and intensive selection for this trait for several decades resulted in a deterioration of many traits like fertility, udder width/circumference and disease resistance (e.g., mastitis, metabolic diseases) traits, etc., and an increase in its ecological footprint (e.g., greenhouse gas emission) (Boichard and Brochard, 2012; Egger-Danner et al., 2015). These factors together with growing demand by consumers for animal safety warrant that successful programs for sustainable animal improvement should create a balance between selection for traits of economic value, animal health, conformation traits, adaptation traits, animal welfare and environmental foot-print.

The successes of genomic selection in Western countries mentioned above were possible through organized and sustained breeding practices supported by government regulations, finance and involvement of private companies. The picture for the majority of African countries is different given that, most livestock are kept for multi-purposes (meat, milk, traction, hides/wool, as a savings account, social status, cultural reasons, etc.), in small herds and flock sizes, under small scale to mid-scale low performing and low input systems, and lack of enabling government policies and financial support. Thus, procedures to increase livestock productivity in Africa in the era of genomic breeding must take into consideration the different production systems, ecological zones and participation of all stakeholders.

### African Livestock Production Systems

In majority of African countries, livestock production is managed under small to large scale systems (Table 3). Small scale production systems include pastoral, agro-pastoral and mixed smallholder farming. Large scale systems include ranching, large scale commercial farming, cooperative farming and state owned farms. About 70% of livestock productivity occurs under the small scale systems characterized by small animal population sizes, low inputs and outputs, etc. Devising appropriate policies for such systems with the right government support is of utmost importance in increasing livestock productivity for food production and income generation. The characteristics of the various systems of livestock production are summarized in Table 3. Under predominantly small scale farming systems, it is important to determine whether or not such systems are ready for genomic breeding. Genomic breeding implementation relies on available genetic resources/diversity, and genomic variation and its association with desired traits.

**Table 3:** African livestock production systems^∗^.

Major category	Production system	Source of labor	Characteristics	Input	Output	Utilization of output	Constraints
Small scale	Pastoral	Small family unit	Transhumance with few to hundred animals, high mortality.	Open communal grazing land shared depending on herd size, manure.	Low production of meat, milk, hide and skin, draft power.	Primarily for home consumption	Recurrent drought, conflict, weak governance, disease, lack of veterinary services and feed resources.
	Agro-pastoral	Pastoral family members	Settled with few to hundreds of animals but migratory for survival, involved in cropping as well.	Open communal grazing land, crop by-products, manure, labor and seeds.	Low production of meat, milk, hide and skin, draft power, crops.	Mainly for home consumption with small portion sold or battered.	Disease, drought, migration of labor to urban areas for jobs, conflict, weak governance, lack of veterinary services and feed resources.
	Mixed smallholder farming	Range from family unit to hired labor depending on herd size.	Small herd size, proximity to markets in urban city, specialization in crop and animal farming.	Crop by-products, household wastes and forage, limited access to inputs such as commercial feeds, manure, fertilizer, improved seeds, irrigation.	Low production of meat, milk, hide and skin, draft power, crops.	Mainly for sale with limited quantity retained for home consumption.	Disease, drought, loss of labor to rural urban migration, conflict, weak governance, limited access to veterinary services and feed resources.
	Urban/ peri-urban	Mainly family unit and hired labor	Small herd-size	Crop by-products, household waste and commercial feed.	Low production of meat and milk.	Home consumption and sale.	Disease, high cost of drugs and feed, and limited water resources and housing.
Large scale	Ranching	Mainly hired herders depending on herd size supported by family members in some instances.	Large herd size, mainly for commercial purpose.	Large rangeland, Veterinary services, drugs, vaccines, supplemented feeds especially during the dry season.	Meat, milk, and hide	Majorly for sale.	Diseases, limited access to veterinary services and land.
	Large scale commercial farming	Hired labor	Access to amenities, infrastructure, organized structure, access to local and export markets.	Veterinary services, drugs, vaccines, feed mill, modern equipment.	Meat, milk, and hide.	Entirely for sale.	Diseases and instability in government policy.
	Cooperative farming	Farm families and also hired labor	Limited access to amenities, infrastructure, local and export markets. Weak organizational structure.	Veterinary services, drugs, vaccines, feeds, few equipment.	Meat, milk, and hide.	Entirely for sale.	Diseases, fluid government policy.
	State owned farms	Civil servants employed by the state	Financed by government, access to local and export markets.	Veterinarians and other livestock professionals, modern equipment and access to government facilities.	Meat, milk, and hide.	Entirely for sale.	Diseases, fluid government policy and bureaucracy.

*^∗^Some of the materials were obtained from Ibrahim (1998), Catley et al. (2016), and Majekodunmi et al. (2016).*

#### African Livestock Genetic Resources, Diversity and Genomic Variation

Effective management of farm animal genetic resources requires adequate information on population size and structure, geographical distribution, the production environment, and within- and between-breed genetic diversity (Groeneveld et al., 2010). Assessment of diversity levels in breeds is necessary owing to husbandry systems which may affect diversity levels through inbreeding and high gene flow between breeds (Ibeagha-Awemu et al., 2004). Information on biodiversity is necessary for preparation of national action plan for improvement of animal genetic resources (Manirakiza et al., 2017). Meanwhile, a consideration for inclusion of genetic information in breed improvement requires knowledge of genomic variation and relationship with traits of interest.

#### Cattle

Africa is home to about 150 cattle breeds distributed across the continent, with the exception of the Sahara and the river Congo basin (Mwai et al., 2015), majority of which are uncharacterized (Nyamushamba et al., 2017). Various categories of cattle are present in the continent including zebu or *Bos indicus* breeds (African humped cattle), taurine or *Bos taurus* breeds (African humpless cattle), hybrids between humpless and humped cattle (e.g., sanga) and sanga and zebu backcross (e.g., zenga). The highest population of cattle and products from cattle are found in the East African region (Table 2 and Figure 1). Clear genetic divergence was revealed between *B. taurus* cattle and zebu breeds of West/Central Africa (Ibeagha-Awemu et al., 2004), and between South African indigenous and locally developed cattle breeds (Makina et al., 2014). However, the breed status of African cattle populations are in danger of disappearing rapidly following uncontrolled crossbreeding and breed replacements with exotic breeds (Ibeagha-Awemu et al., 2004; Mwai et al., 2015; Traoré et al., 2017).

Using microsatellite markers, candidate gene and genome wide approaches, genomic variation in some African cattle populations have been assessed and in some cases associated with production traits. Using 28 autosomal markers, Ibeagha-Awemu et al. (2004) revealed that zebu breeds in Cameroon and Nigeria are highly diverse as well as closely related. Whole genome SNP panel indicated close relationships between South African indigenous and locally developed cattle breeds (Makina et al., 2014) as well as pure and crossbred cattle in Burundi (Manirakiza et al., 2017). Genome characterization by sequencing of five indigenous African cattle breeds representatives of the cattle diversity of the continent [namely N’Dama (West African taurine), Ankole (African sanga cattle), Boran (East African zebu), Kenana (East African zebu), and Ogaden (East African zebu)] revealed a high number of SNPs in the breeds as well as breed specific SNPs (Kim et al., 2017). On a genome-wide window scale of 10 Mb, all indigenous African breeds had higher levels of nucleotide diversity compared to commercial European breeds (Angus, Jersey, and Holstein) which have been subjected to intensive artificial selection over generations (Kim et al., 2017). Genome wide characterization with Illumina BovineHD or BovineSNP50 Genotyping BeadChip of cattle breeds from East Africa, North Africa, South African, and West Africa revealed positional candidate positive selection regions which encompass genes and quantitative trait loci (QTL) for milk traits, reproduction and environmental stress (immunity and heat stress), candidate genes associated with biological pathways important for adaptation to marginal environments such as immunity, reproduction, development, and heat tolerance, copy number variations enriched for a number of biological processes, molecular functions and cellular components as well as potential to improve some of the breeds for dairy traits through breeding (Bahbahani et al., 2015, 2017, 2018; Pierce et al., 2018). Moreover, footprints of adaptive selection at the whole genome level (genotyping with 36,320 SNPs) were identified in nine West African cattle populations, including 53 genomic regions and 42 candidate genes enriched in physiological functions such as immune response, nervous system, and skin and hair properties (Gautier et al., 2009). From these data, high levels of genetic diversity is evident within African cattle populations which have been attributed to domestication, long history of migrations, selection and adaptation (Luikart et al., 2001; Groeneveld et al., 2010; Kim et al., 2017). Due to exposure to strong environmental pressures (hot, dry, or humid tropical climate conditions), diverse disease and nutritional challenges and water shortages, African livestock populations display unique adaptive traits (Table 4) which are necessary to support productivity and survivability in the different ecological zones.

**Table 4:** Adaptive characteristics of some African livestock breeds.

Breed	Species	Character	Main location	References
West African Dwarf	Goat	Trypanotolerant, resistant to gastro-intestinal parasites, prolific, good kidding interval	West Africa	Review by Kosgey et al. (2006)
Red Sokoto	Goat	High quality skin	West Africa	“
Galla	Goat	Trypanotolerant	Kenya/East Africa	“
Mubende	Goat	High quality skin	Uganda	“
Nubian	Goat	High milk yield	The Sudan	“
Small East African	Goat	Trypanotolerant, resistant to gastro-intestinal parasites	Kenya/East African	“
West African Dwarf goat	Goat	Trypanotolerant	West Africa	Faye et al., 2002
West African Dwarf sheep	Sheep	Trypanotolerant,	West Africa (humid and sub-humid areas)	“
D’man	Sheep	Fecundity	West Africa	“
Blackhead Persian	Sheep	Trypanotolerant, heat tolerant	East Africa	“
Red Maasai	Sheep	Trypanotolerant, resistant to gastro-intestinal parasites	East Africa (humid and sub-humid areas)	“
West African Dwarf sheep	Sheep	Trypanotolerant	West Africa	Geerts et al., 2009
Muturu	Cattle (humpless short horn cattle)	Trypanotolerant	Nigeria	Adebambo, 2001
N’Dama	Cattle (humpless long horn cattle)	Trypanotolerant, tolerant to cattle ticks	West Africa	O’Gorman et al., 2009
N’Dama	Cattle (humpless long horn cattle)	Trypanotolerant, tolerant to cattle ticks	Central and West Africa	Mattioli et al., 2000
Ankole	Cattle (humpless long horn cattle)	Tolerant to cattle ticks	West Africa	“
Doayo (*Bos taurus*)	Cattle	Trypanotolerant	Cameroon	Achukwi et al., 2009
Taurine × Zebu crossbred	Cattle	Trypanotolerant	Burkina Faso	Dayo et al., 2011
Sheko	Cattle (humpless short horn cattle)	Trypanotolerant	Ethiopia	Lemecha et al., 2006
Orma Boran	Cattle (large East African Zebu)	Trypanotolerant	Kenya	Njogu et al., 1985; Maichomo et al., 2005
Nuba Mountain Zebu	Cattle (small East African Zebu	Trypanotolerant	Sudan	DAGRIS, 2007
Azaouak	Cattle (West African Zebu)	Adapted to drought	Niger, Nigeria	DAGRIS, 2007
Landim	Cattle (South African Sanga)	Resistant to foot and mouth disease	South Africa	Reviewed by Mwai et al., 2015
Tswana	Cattle (South African Sanga)	Resistance to endemic heartwater, tolerance to ticks	South Africa	Asselbergs et al., 1993
Red Fulani	Cattle (West African Zebu)	Trypanotolerant, good beef characteristics	West Africa	Mamoudou et al., 2016; Bayemi et al., 2015
White Fulani	Cattle (West African Zebu)	Good dairy and beef characteristics	West Africa	Pullan and Grindle, 1980; Etela et al., 2008
Raya-Azebo	Cattle (East African Sanga)	Good draft power	Ethiopia	DAGRIS, 2007
Indigenous	Pig	Tolerance to African swine fever	Western Kenya	Mujibi et al., 2018
Indigenous	Pig	Descent growth rate, good meat quality, decent litter size, low feed cost	South Africa	Madzimure et al., 2013
Ashanti Dwarf	Pig	Hardy with disease resistant traits	Ghana	Osei-Amponsah et al., 2017
Indigenous	Chicken	Disease, drought and heat tolerance	Rwanda	Mahoro et al., 2018
Indigenous	Chicken	Disease and stress tolerance, good egg hatchability and good meat taste	Ethiopia	Dana et al., 2010
Indigenous	Chicken	Heat tolerance	Kenya	Kennedy, 2016
Ecotypes	Chicken	Tolerance to environmental stress	Uganda and Rwanda	Fleming et al., 2016

#### Goat

The domestic goat, *Capra hircus*, is an important livestock species that is well suited to small-holder production systems throughout the entire African continent. Unique to West Africa is a great genetic diversity of goat types; the long-legged and trypano-susceptible types (e.g., Sahel and Red Sokoto goats) found in tsetse free areas and the trypano-tolerant type (West African Dwarf goat) found in the humid zone. According to Missohou et al. (2011), different ecotypes have emerged under varying selection pressures and diversified climate and topography in different countries. The largest goat populations are found in the Eastern and Western African regions (Table 2). Genetic diversity study on African goats is generally limited compared to other continents (Groeneveld et al., 2010). Microsatellite studies revealed a substantial amount of within breed diversity based on mean number of alleles observed (Muema et al., 2009; Missohou et al., 2011; Traore et al., 2012; Murital et al., 2015). Using genome-wide SNP data, Mdladla et al. (2016) reported high level of genetic diversity in South African indigenous goats including three locally developed meat type breeds of Boer, Savanna and Kalahari Red, a feral breed of Tankwa and unimproved non-descript village ecotypes. Some African goats have been characterized for polymorphisms in genes that control economically important traits (milk traits and litter size) (Bemji et al., 2006; Missohou et al., 2006; Caroli et al., 2007; Isa et al., 2017; Bemji et al., 2018), pointing to their potential application for genetic improvement for these traits.

#### Sheep

Diverse populations of sheep are found in the African continent with about 170 breeds of domestic sheep found in sub-Saharan Africa (Kemp et al., 2007). Present-day African sheep population is about 352 million (FAOSTATS, 2018) out of which ∼62% are found in Northern and Western Africa (Table 2). Investigations from different African countries based on microsatellite markers (Gaouar et al., 2015), mitochondrial DNA (Agaviezor et al., 2012; Brahi et al., 2015) and genome-wide SNP chip (Edea et al., 2017) revealed high within breed than between breed genetic diversity with clear evidence of admixture between breeds of sheep. The latter authors further observed that North African sheep breeds showed higher levels of within-breed diversity but were less differentiated than breeds from Eastern and Southern Africa, confirming previous reports that sheep from South Africa showed low to moderate genetic diversity (Qwabe et al., 2013). The initially domesticated sheep breeds in West Africa have also been genetically mixed with European breeds (Brahi et al., 2015). Using the OvineSNP50 beadchip, Molotsi et al. (2017) reported that the smallholder Dorper sheep was introgressed with Namaqua Afrikaner, South African Mutton Merino and White Dorpers genes. They further reported that the smallholder Dorper population was more genetically diverse than the pure-bred Dorper, South African Mutton Merino and Namaqua Afrikaner. Sheriff and Alemayehu (2018) reported low observed and expected heterozygosity in Ethiopian, Kenyan, South African and Nigerian sheep populations. They opined that the low heterozygosity may be due to the effect of small population sizes, inbreeding and minimal or null immigration of new genetic materials into the close populations. These data suggest close relationships and high levels of genetic admixture between African sheep breeds, especially among populations in the same geographic area.

#### Chicken

The domestic chicken with an estimated population of more than 1.9 billion in 2016 (FAOSTATS, 2018) is the most common and widespread domestic animal species kept mainly for food (meat and eggs) by resource poor farmers in Africa. Large-scale analyses involving microsatellite loci in domestic chickens, commercial lines and chickens sampled from the European region revealed high mean numbers of alleles and high degree of heterozygosity in Asian and African chickens as well as in Red Jungle fowl (Lyimo et al., 2014). Lower degree of population stratification as well as high within-breed genetic diversity in African chickens are supported by analyses with microsatellite markers (Muchadeyi et al., 2007; Adebambo et al., 2010; Mtileni et al., 2011), mtDNA (Wani et al., 2014; Hassaballah et al., 2015; Eltanany and Hemeda, 2016) and genome-wide SNP chips (Khanyile et al., 2015a,b; Fleming et al., 2016, 2017). Reduced genetic diversity was, however, witnessed with conservation flocks in South Africa which represented a limited sample of the gene pool (Muchadeyi et al., 2007; Mtileni et al., 2016). Increasing expansion of the commercial chicken industry and intermixing of commercial hybrids with local strains in rural backyards are eroding the genetic uniqueness of native breeds and their potential to adapt to local conditions (reviewed by Eltanany et al., 2011). Lawal et al. (2018) reported the use of whole-genome resequencing data of Red Jungle fowl and Indigenous Village Chicken populations from Ethiopia, Saudi Arabia, and Sri Lanka to decipher regions of the genome with functions relating to adaptation to temperature gradient, reproduction and immunity. All these results indicate the presence of genetic variation that can be utilized in genomic breeding.

#### Pig

The local African Pig is small in size and is likely the same breed in all African countries known under various names (African Union Inter-African Bureau for Animal Resources [AU-IBAR], 2015a), such as: Kolbroek (South Africa), Somo (Mali), Bakosi (Gabon and Cameroon), West African Dwarf pig (Nigeria), Ashanti Dwarf pig (Ghana), Bush pig (Togo), Mukota pig, or Zimbabwe Mukota pig (Zimbabwe). Despite cultural and religious influences in parts of the continent that limit pork production and consumption, pig farming is generally growing across West, East, Central and Southern Africa (African Union Inter-African Bureau for Animal Resources [AU-IBAR], 2015a) with the highest populations in Eastern and Western Africa (Table 2).

Findings based on joint analysis of mitochondrial, microsatellite and Y-chromosome polymorphisms in pigs and wild boars with a worldwide distribution revealed remarkably weak genetic differentiation between pigs and wild boars (Ramirez et al., 2009). This was attributed to a consequence of a sustained gene flow between both populations. More recent findings on pig populations indigenous to southern Africa based on different microsatellite loci (Halimani et al., 2012) similarly revealed lack of substructure in the pig populations, corroborating the general similarity in phenotypes commonly reported (Halimani et al., 2012). Sampled pigs in Ghana represented distinct populations with a moderate amount (12%) of genetic differentiation (Ayizanga et al., 2016). A study on the estimation of genetic parameters for growth performance and carcass traits in Mukota pigs in South Africa reported the presence of sufficient genetic variation that can support genetic improvement for many growth and carcass traits in the breed (Chimonyo and Dzama, 2007). Using the porcine genome wide SNP chip, Mujibi et al. (2018) observed a significant introgression of genes from international commercial breeds into Busia pigs from Busia county in Kenya. The authors also reported that pigs from Homabay county in Kenya are distinct from the international breeds and thus represent a local indigenous gene resource.

## Considerations and Strategies for Implementation of Organized Genomic Breeding in Africa

**FIGURE 2:**
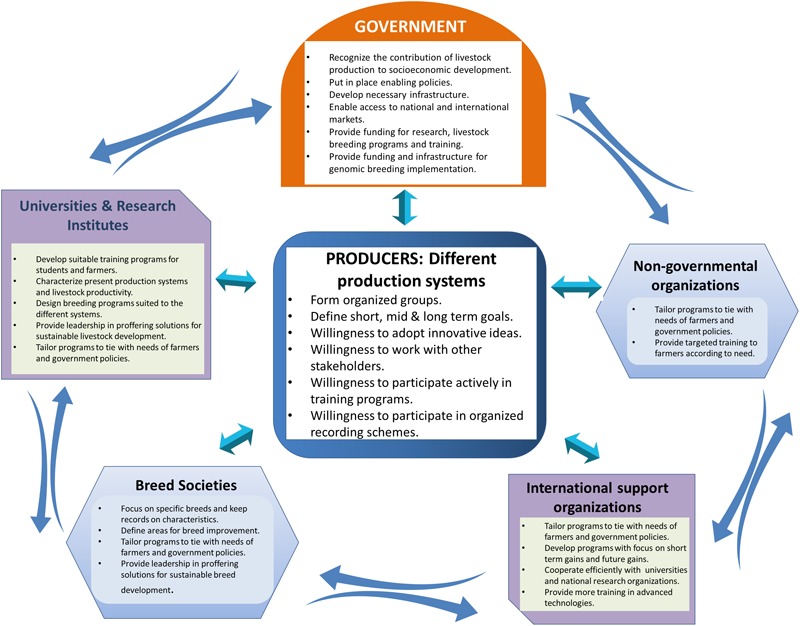
Different stakeholders that should be harnessed for successful livestock breeding programs in Africa.

For successful implementation of structured genomic breeding programs for African livestock populations, several factors deserve consideration as well as collective action and cooperation by all stakeholders (farmers, governments, research professionals, research organizations, universities, breed societies, private businesses, and support organizations) working together to achieve a common goal as illustrated in Figure 2.

### Availability of Genetic Material for Breeding

As discussed above, the African continent is home to diverse livestock populations which also display rich genomic variations within and between breeds and have acquired special adaptive characteristics that support adaptation to poor quality feed, limited water supply, hot environments and disease (Psifidi et al., 2016; Mrode et al., 2018; Lawal et al., 2018). Some of these characteristics are summarized in Table 4. The indigenous breeds have acquired important characteristics for survival in their environments and in addition to being developed systematically, should be conserved for future survival and exploitation. Therefore, conservation of local breeds (highly utilized and less utilized) must be part of national breeding plans and should not be an exercise undertaken by individual farmers. Systematic breed development strategies including selection within breeds, controlled crossbreeding and upgrading programs and development of new breeds to exploit special adaptive and/or production traits must be done under organized systems with specific goals. This will address the practice of indiscriminate crossbreeding between local breeds and, between imported breeds and local breeds that is eroding the continent’s animal genetic diversity, a much needed resource for present and future exploitation.

### Understand Production Systems, Production Potentials of Livestock and Needs of Farmers

There is still a lack of understanding about situating livestock development programs within prevailing low-input production systems, societal preferences and environmental conditions. Most international development programs have been based on ‘top-down approaches,’ considering single commodities and technology focused orientation with little or no participation of farmers nor formation of strong farmer-based institutions (Kaasschieter et al., 1992) and with no regard to prevailing environmental conditions. Majority of livestock are produced under the small holder system composed of pastoralists, agro-pastoralist and small holder farm families which have different breed preferences, inputs and challenges. Therefore, the prevailing production systems and livestock production potential under the different systems must be characterized and their specific needs identified in relation to local preferences and market needs, with the participation of producers. Data on productivity of local breeds under their prevailing conditions of production are largely unavailable. Such data is necessary as it will form the basis for improvement plans for each system. Indigenous African breeds are generally considered as underproductive without giving thought to the low-input and harsh environments in which they are raised. For example, under farmer management, the Butana and Kenana zebu breeds of Sudan produce averagely 538.26 and 598.73 kg of milk per lactation, respectively, while under research station conditions, they produce 1,400–2,100 kg of milk per lactation, respectively (Musa et al., 2005, 2006; Yousif and El- Moula, 2006). This implies that, although Butana and Kenana seem to produce less under low-input systems, they have the ability to produce more given improved conditions of nutrition, health care and production management. Another factor is the bias in judging local breeds based on parameters that have been selected and developed in exotic breeds. An example is the focus on lean specialized pig breeds like Large White at the expense of local relatively fat breeds that are well adapted to local environmental conditions. As such, policies are put in place that disfavor production of local pigs but encourage their replacement with exotic breeds (Chimonyo and Dzama, 2007).

Furthermore, emphasis has been placed on realizing quick gains by adopting ‘shortcut approaches’ like introduction of exotic genes that have been developed over a long period of time in different environments. This exotic stock does experience genotype by environment interaction which dramatically lowers performance in the local environment where they are introduced. African livestock have acquired the characteristics necessary to produce under their prevailing environmental conditions and on minimal resources. Before introducing exotic genes in a controlled manner, firstly, the productivity of local breeds must be assessed under optimal conditions (e.g., adequate feeding, housing, and disease management). The main limiting factors of local breed productivity could just be management and limited feed resources and disease control measures, as exemplified in Butana and Kenana cattle (Musa et al., 2005, 2006; Yousif and El- Moula, 2006). Under optimal management conditions, local breeds could be selected for desired traits in their prevailing environmental conditions. The adoption of most ‘shortcut approaches’ utilizing exotic genes has generally not resulted in substantial gains and sustainable long-term increases in productivity or contributed to poverty alleviation. Most donors or policy makers are only interested in immediate short term gains (visible) with the result of reckless crossbreeding of indigenous cattle resources with exotic breeds or their complete replacement with exotic breeds. These ‘short term gains’ are usually lost when such programs end and usually, the offspring of crossbred animals underperform under the prevailing conditions or lose the adaptive productive ability of the local breeds.

### Breeding Goals

Setting a clear breeding goal is a prerequisite for animal improvement planning and implementation of genomic selection. A definition for animal breeding goals planning as a procedure with ethical priorities and weighing of market and non-market values has been suggested (Olesen et al., 2000). The decision to develop an animal for specific products has primarily been for the common interest of the farmer or the society or market demand. For example, increased market demand for milk and its products drove dairy breeding objectives in Western countries toward increased milk yield which unfortunately has resulted to problems of fertility and huge environmental footprints (Gill et al., 2010; Wall et al., 2010; Hayes et al., 2013; Knapp et al., 2014). Hence, sustainable animal breeding goals should consider market economic and non-market value traits, farmer specific needs, social, ecological and environmental needs supported by appropriate government policies, education, more cooperation between stakeholders and, short and long term needs, etc. The breeding goals must be adapted to fit each production system and environment. In recent times, breeding goals considering new phenotypes or non-traditional or production oriented traits and genetic traits of relevance to breeding sustainability have been proposed for cattle, sheep and pigs (Merks et al., 2012; Banga et al., 2014; Miglior et al., 2017; Molotsi et al., 2017). Recently, results of a survey of 160 farmers in southern Mali identified draft power and savings as the most important production objectives while preferred traits included fertility, draft ability and milk yield, in that order (Traoré et al., 2017).

### Feed Resources and Animal Health

Optimal animal productivity is supported by adequate nutrition and disease management. Options for quantitative and qualitative improvements of the feed resources according to the needs of the different production systems are required for sustainable livestock systems (Duncan et al., 2013; Thornton and Herrero, 2015). For example, under the pastoral system, communal access to rotational grazing pastures and fodder banks which should be maintained to ensure quality of feed resources will support sustained livestock production. Legislations instituting the development of watersheds, restrictions on indiscriminate burning of grazing land and use of such land for other purposes are of necessity. Other vital aspects include development of improved pastures and fodders, increased grain production, development of agricultural bi-products as feed resources, access to water resources, etc. Although some of the local breeds have adapted to the disease burdens of their environments, disease is still a major limiting factor to livestock productivity in the region (De Garine-Wichatitsky et al., 2013; Okuni, 2013; Vanderburg et al., 2014). Particular attention should be paid to disease control measures like access to drugs, vaccines and veterinarians, and sound management practices developed for each system (Maclachlan and Mayo, 2013; Miguel et al., 2013).

### Data Acquisition

Precise phenotypic data is crucial for genetic improvement. In Western countries, systems have been put in place to support high throughput phenotyping (e.g., milk yield, milk component yields, feed intake, etc.) thus enabling the accurate and consistent collection of large amounts of data on animal productivity. The formation of livestock trade databases is worthy of consideration since livestock movement contributes to the spread of animal and zoonotic diseases. In Western countries, this database is important for researchers to describe mobility patterns, optimize disease surveillance and control and predict possible epidemic scenarios (Apolloni et al., 2018). Therefore, it is necessary to sensitize producers on the importance of data collection and record keeping on the productivity of their animals, as well as formation of data storage facilities that can facilitate data storage and sharing within and between countries.

### Infrastructure and Environmental Considerations

Besides the common issues with infrastructure for general development of the economy, infrastructural development to promote livestock production within the continent must be considered such as basic equipment for sample storage, data collection and data trace, livestock markets, slaughter facilities, animal housing and pasture development. With advances in genomics and other omics technologies, the livestock sector in several countries has moved to the area of big data research and application (VanderWaal et al., 2017; Morota et al., 2018; White et al., 2018). African livestock infrastructure must be developed to optimize the use of big data. Moreover, farmers need to be sensitized and prepared for adoption of these technologies. Technologies need to be adapted to farmers’ specific needs according to the system of production since there are differences in farmers’ access to farm resources, technological inputs and differences in access to output markets (Birhanu et al., 2017; Feder and Savastano, 2017). For instance, adapted dairy technologies varies widely among smallholders (Staal et al., 2002; Abdulai and Huffman, 2005; Amlaku et al., 2012) and also strongly affected by their social networks (Amlaku et al., 2012). The environmental impact is now a major concern for livestock production the world over due to its impact on greenhouse gas emissions and consequently climate change. The livestock sector in Africa also pose a challenge to the environment and climate change depending on the management and farming system. For instance, the semi-arid region is faced with the problem of overgrazing of rangelands which is caused by population pressure and a decline in traditional management systems (Fratkin, 2001; Ngongoni et al., 2006).

### Development of National and Regional Policies and Priorities That Support Effective Production and Utilization of Livestock

The success of sustainable livestock development in any country or region hinges on development of national and or regional policies or guiding principles in the conduct of affairs. The decision by an international support organization or by a farmer group to import specific germplasm for crossbreeding with local breeds must be backed by national polices and priorities. Supply and demand policies favoring local production and supply chains will stimulate local production. Recognizing the important contribution of livestock production to the livelihood of farm families and to the nutrition and economy of the state/country necessitates a political commitment to stimulate, develop and financially sustain livestock development. The African Union has in place a Livestock Development Strategy (LiDeSa) for Africa (2015–2035) which was developed through an inclusive consultation process involving experts and stakeholders at national, regional, and continental levels (African Union Inter-African Bureau for Animal Resources AU-IBAR [AU-IBAR], 2015b). The strategy recognizes the central role played by livestock as a livelihood sustainer for rural Africa and with the support of a grant from the Bill and Melinda Gates foundation, seeks to transform the livestock sector by invigorating its untapped potentials. This is a laudable process that if implemented could truly transform lives. However, country level initiatives must follow suit for a transformed livestock sector to emerge in the continent. Today, the South African government is the only African government that is playing an active role in the conservation of animal genetic resources (Nyamushamba et al., 2017) and in supporting livestock breeding programs (Van Marle-Köster et al., 2017). Moreover, it is also important to take into account farmer’s preferences in the development of breeding polices (Wale and Yalew, 2007). Development of national policies and regional priorities should also focus on mitigation in the livestock sector due to the impact of climate change which varies with location. A program called climate-smart agriculture (CSA) has been implemented recently in the West African region and sub-Saharan Africa in general (Amole and Ayantunde, 2016). CSA is an approach that provides a conceptual basis for assessing the effectiveness of agricultural practice change to support food security under changing climatic conditions (Amole and Ayantunde, 2016).

### Creation of Markets and Facilitation of Access to Markets

Appropriate economic incentives are important for livestock genetic improvement. Breeding programs should be market-oriented and the government should provide the right incentives. Several countries have made efforts on the extension of market access as well as to encourage foreign trade. For example, the Ethiopian government has completely strategized to encourage foreign trade for sheep and goat products which has led to the creation of employment opportunities for its citizens (Nwogwugwu et al., 2018) or the emergence of livestock feed market in Ghana (Konlan et al., 2018).

### Education and Training, and Information Sharing

Education and training, and information sharing are vital aspects in sustainable livestock improvement breeding. The training curriculum in higher institutions should be adapted to fully address the needs of the various production systems. Formal training of students and informal training of the producers is vital. Greater cooperation between universities, research organizations (international, national, and regional), producer groups, non-governmental organizations and governments will ensure the flow and sharing of information and knowledge (Figure 2). The International Livestock Research Institute (ILRI) in Kenya has and continues to train students, research professionals and farm groups in various aspects of livestock breeding and production and molecular biology/genomics techniques. ILRI’s work in consultation with and tailored to meet the needs of farm families has resulted to initiatives like the Dairy Genetics East Africa project (DGEA), African Dairy Genetic Gains (ADGG), etc^3^. These programs were tailored to increase farmer productivity and profitability through the use of cross-bred animal types supported by extension and training systems tailored to their needs. The influence of ILRI amidst other successes led to rapid increase in cow milk production between 2011 and 2012 in the East African region (Figure 1). A national milk recording scheme has been instituted and supported by the government of Kenya^4^. The challenges faced by the program include limited number of breed inspectors, unawareness by many farmers of the importance of livestock registration, delay in issuance of livestock certificates and poor record keeping by farmers. Some of the suggested solutions include: training of more livestock inspectors by breed societies in conjunction with government, create farmer awareness using sensitization campaigns through mass media, exhibitions, shows, field days and direct consultations with interested farm groups, decentralization of services and investment in manpower and infrastructure. National animal production research institutes and universities in the various countries can emulate some of the practices of ILRI given that farmer’s participation in the development of projects tailored to their needs is a vital aspect in the successes of such programs.

Regional and continent wide sharing of information is vital for the sustainability of the livestock sector. The Forum for Agricultural Research in Africa^5,6^, a technical arm of the African union, coordinates and advocates for agricultural research-for-development in the continent. Regular meetings of stake holders (professionals, farmers, students, and industry) interested in the act of animal production in forums like the All African Conference on Animal Agriculture, country and regional conferences on animal production all serve vital roles in the flow of information and technology advancements. However, producer focused meetings that provide informal training to farmers are generally lacking.

## Application of Modern Genomic Breeding Technologies in African Livestock

Rapid improvement of African livestock productivity can benefit from current modern breeding technologies but many limitations abound. Some breeding programs that have been implemented for genetic improvement of livestock in Africa and the challenges faced are summarized in Table 5.

**Table 5:** Sample breeding programs for genetic improvement of livestock in Africa.

Breeding system	Characteristic/objective	Species	Region	Advantage	Disadvantage	References
Selection within breed or strain	Selection based on productivity in individual populations for litter size, growth and mature size etc.	Small ruminants	Tropics	Increase average level of genetic merit of the population; Less costly compared to selection between breeds	Small populations; single sire flocks; Lack of animal identification; inadequate performance and pedigree recording; high mobility of pastoral flock.	Review by Kosgey et al. (2006)
Selection between breeds or strains	Selection of most appropriate of two or more genetically distinct breeds	Small ruminants	Tropics	Can achieve rapid genetic change when there are large genetic differences between populations	Involves high cost when there is need to replace males and females (not always feasible to replace whole flock).	Kosgey et al., 2006
Crossbreeding	Grading up or repeated crossing to a new breed using males or semen	Small ruminants	Tropics	More gradual improved genetic changes in desired traits	Unsuccessful and unsustainable in long term due to incompatibility of genotypes with breeding objectives and management approaches of the prevailing low input traditional production systems.	Review by Onteru et al. (2010)
“	Market oriented dairy goat farming	German Alpine × Kenyan local goat breeds	Kenya	Improved average daily milk yield	Lower survival rate for crossbred.	Bett et al., 2011
“	Dairy farming (cattle)	*Bos Taurus* ×*Bos indicus*		Improved milk yield	Crossbreds are more susceptible to diseases.	Review by Onteru et al. (2010)
“	Dairy farming (cattle)	Ankole × European cattle	Burundi	Milk yield increased in crossbreds with increasing European ancestry	Not reported	Manirakiza et al., 2017
“	Large body size, fertility, drought ability and milk yield	Ndama × Fulani Zebu and crossbred	Southern Mali	Disease resistance for Ndama, high market price for Fulani Zebu and crossbred cattle	Not reported	Traoré et al., 2017
“	Trypanotolerance	Taurine × Zebu cattle	South Western Burkina Faso	Increased anemia control by crossbred cattle	Not reported	Dayo et al., 2011
Terminal cross-breeding	To demonstrate the performance of Nguni and Afrikaner as dam lines	Cattle (Nguni and Afrikaner)	South Africa	Reduced calving difficulty, increased survival percentage from calving to weaning, the ratio of the weaning weight of the calf to the dam weight at the birth of the calf was 56.8% for cross-bred	Limitation in calving difficulty and birth weight restricted to mid-parent value or below.	Scholtz and Theunissen, 2010
Traditional village breeding	Organize smallholder farmers into cooperative breeding groups for genetic improvement	Sheep (Menz)	Ethiopia	Annual increase in body weights at birth, 3 and 6 months of age were 0.004, 0.11, and -0.12 kg, respectively. The genetic gain in 6-month weight of lambs was 0.92 kg.	Uncontrolled mating, random selection, low selection intensities. There is stagnating or declining trends in body weights.	Gizaw et al., 2014b
Cooperative village breeding	Optimization of a cooperative village-based sheep breeding scheme	Sheep (Menz)	Ethiopia	The genetic gain in 6-month weight of lambs was1.54 kg. Allows farmers’ cooperation and leads to genetic improvement	Efficiency of controlled mating was 0.75. Small flock sizes. Difficulty’ in actively getting farmers involved in cooperative village breeding. Selection intensity reduces with declining membership because of smaller flock sizes. With uncontrolled mating, percentage of the village ewes available for mating by unselected rams increased as the level of participation in the selection program reduced. Genetic progress dropped with declining participation of villagers.	Gizaw et al., 2014a
Crossbreeding	Determination of better performing breed and breed combination	Goat (Mubende and Teso)	Uganda	Crossbreds had higher body weight and growth rate relative to the pure breeds	Not reported	Ssewannyana et al., 2004

Livestock in the African continent are highly adapted to the prevailing environmental conditions characterized by heavy disease burden and marginal feed resources, but with marginal productivity because they are still largely unselected. African countries can benefit from genomic selection because it could be done even without pedigree information which is essential to traditional best linear unbiased prediction (BLUP)-EBV and the selection of candidates does not necessarily have to be based on trait records. The potential to generate GEBV using molecular information makes genomic selection a very attractive alternative to improving livestock in developing countries where adequate phenotypes and pedigree records are lacking. Genomic breeding has been reported to be more accurate than traditional BLUP because genomic relationships are more accurate than pedigree relationships (Meuwissen et al., 2016). Moreover, understanding of the fundamental genetic mechanisms influencing traits can be useful for setting up priors for (genetic) variances to increase the accuracy of genomic selection. Several successful approaches have been introduced such as BLUP| GA (BLUP-given genetic architecture; Zhang et al., 2014) or BayesRC (which adapted BayesR methods) incorporating prior biological information in the analysis by defining classes of variants likely to be enriched for causal mutations (MacLeod et al., 2016) or single step GBLUP with prior information (Fragomeni et al., 2017). These methods can be particularly useful for genomic selection in Africa with some prior biological knowledge of traits obtained from studies in the populations and other populations. Using genomic selection, Pitchford et al. (2017) concluded that heterozygosity effects were substantial for reproduction and growth in a tropically adapted composite beef program.

Our high enthusiasm about the potential application of genomic selection in African countries is immediately dampened with the reality that animals are held in small populations and in many small holder units. Furthermore, male animals that drive the genetic gain are often sold to generate income for farm families. These caveats can be overcome by the formation and practice of communal management and breeding systems.

Lack of phenotypes recorded in accurately defined contemporary groups is one of the constraints to the implementation of genomic selection in Africa and many developing countries (Burrow et al., 2017). Acquiring the genomic information for genomic selection is limited because genotyping is still expensive in many developing countries because incomes are very low compared to developed countries. The few studies on genomic selection in developing countries are characterized by small population sizes and validations were undertaken with test day data sets (Neves et al., 2014; Brown et al., 2016; Kariuki et al., 2017; Ducrocq et al., 2018; Mrode et al., 2019).

Traditional animal breeding requires the use of pedigree records to support selection decisions but most small holder farms in Africa do not have these types of records and the measure of relationships between animals are merely speculative. Furthermore, the application of genomic selection will require the use of reference populations which are generally lacking in Africa and many developing countries (Burrow et al., 2017). Mrode et al. (2019) reported the presence of small reference populations of between 500 and 3,000 animals (composed of mostly cows) in dairy and beef cattle in developing countries. The use of small reference populations that combined both bull and cow data, as in the case in Africa, has implications for the accuracy of genomic prediction, which is lower when compared to those obtained in Western countries given the limited information of the response variables when using cow records. It is important to state here that the inclusion of cows in the reference population has resulted to up to fivefold increase in the size of the reference population in some cases and increases of up to 12% in accuracy of selection compared to using bulls alone (Boison et al., 2017; Mrode et al., 2019). Mrode et al. (2018) reported some success by modeling and pooling data on the accuracy of genomic prediction in limited dairy data in East Africa. Brown et al. (2016) specifically reported the practice of genomic selection in a crossbred cattle population using data from the dairy genetic project of East Africa.

The cost of genotyping is a major issue limiting the adoption of genomic selection in Africa and to overcome this problem, the use of low density SNP panels have been suggested and this can be followed with imputation to improve the accuracy of genomic predictions (Meuwissen et al., 2016; Boison et al., 2017). Furthermore, low cost genome wide genotyping solution like genotyping-by-sequencing can generate high numbers of population specific SNPs (De Donato et al., 2013; Ibeagha-Awemu et al., 2016; Gurgul et al., 2018) that can support genomic selection in African livestock populations. Illumina^7^ and Affymetrix^8^ commercial SNP panels used for genotyping contains SNPs discovered in breeds and population of animals of Western origin and only very few breeds of African origin were included in the discovery of SNPs. This is the reason for ascertainment bias, which may affect accuracies of genomic selection from the use of commercially available SNP panels to genotype African indigenous livestock. Thus, the development of genotyping solutions specific for African breeds is necessary and the genotyping-by-sequencing approach can play a major role.

Some notable developments in the use of genomic tools include the sequencing of some indigenous cattle in Africa (Kim et al., 2017), developments on the genomic selection for disease resistance (Hanotte et al., 2010; Mwai et al., 2015) and for adaptation to hot arid condition (Kim et al., 2016). Other important efforts that may increase the quality of data includes the project of epidemiology of the Infectious Diseases of East African Livestock and a longitudinal calf cohort study in western Kenya (de Clare Bronsvoort et al., 2013) and strategies for bridging the gap between the developed and developing livestock sector (Van Marle-Koster and Visser, 2018). Recently, Canovas et al. (2017) discussed the application of new genomic technologies including transcriptomics, metagenomics, metabolomics, and epigenomics that are pertinent to speed-up genetic improvement of cattle. As a matter of priority, Burrow et al. (2017) suggested that research to improve grazing livestock should include cross-country genetic/genomic evaluations, use of sequence data in genetic evaluations, multi-breed genomic evaluations, selection index and genotype × environment interactions. Furthermore, numerous studies in Nellore, an indicine beef cattle breed suggests that genomic selection is a realistic alternative to traditional selection strategies (Neves et al., 2014). In small ruminants like sheep and goats, Mrode et al. (2018) observed that innovative genetic selection strategies will be needed to ensure adaptive balance between production and adaptation.

Emerging gene editing technologies like transcription activator-like effector nucleases (TALEN), zinc finger nucleases (ZFN), and clustered regularly interspaced short palindromic repeats (CRISPR)/Cas9 which can achieve any change in the genome, including introduction of alleles of interest into a recipient genome and switching on/off genes of interest can also play vital roles in rapid genomic improvement of African livestock traits. These tools offer an opportunity to intensify the frequency of desired alleles in a population through gene-edited individuals more rapidly than conventional breeding (Bhat et al., 2017). Genome editing in livestock has been reported for the double muscling gene in cattle, sheep, and pigs (Proudfoot et al., 2015; Qian et al., 2015), the polled allele introduction in dairy cattle (Tan et al., 2013; Carlson et al., 2016); gene edits that confer resistance to African Swine fever virus in pigs (Lillico et al., 2013; Whitworth et al., 2016) and the low-density lipoprotein receptor gene in a pig model of atherosclerosis (Carlson et al., 2012). These examples indicate that attempts at gene editing in livestock have targeted traits controlled by few variants with major effects. However, most livestock traits of economic importance are quantitatively controlled by many genes each contributing small effects, suggesting potential pitfalls in the implementation of these technologies for such traits. However, a recent simulation study indicated that editing for fewer casual variants of polygenic traits can double the rate of both short term and long term genetic gains when compared to conventional genomic selection (Jenko et al., 2015).

## Challenges and Way Forward

As mentioned above, most countries in Africa lack functional breeding programs due to lack of involvement and engagement of farmers or producers and other stakeholders. Therefore, it is important to have long-term plans for breeding programs, which can meet present and anticipated future market needs (Zonabend et al., 2013). The major constraints to implementation of genomic breeding approaches for African livestock populations and the way forward have been discussed in Section “Considerations and Strategies for Implementation of Organized Genomic Breeding in Africa” and summarized in Table 6 and the major roles of each stakeholder are summarized in Figure 2.

**Table 6:** Major concerns and possible solutions for development of improved livestock breeding programs in Africa.

Constraint	Way forward
Small herd sizes with single sires; lack of proper identification; variability between farms; uncontrolled breeding and high mobility of pastoral flock.	Organized livestock production systems: cooperative farming; formation of national breed societies with regulatory agencies.
High disease burden, limited access to drugs and veterinary services.	Government should train veterinarians and provide free/subsidized veterinary services and medication. Increase/provide funding for research on livestock diseases.
Limited feed resources to meet nutrient requirements of animals.	Develop feed resources and grazing pastures and train animal nutritionist, animal feed producers and farmers.
Limited information on production characteristics of indigenous breeds under existing production systems.	Systematic characterization of animal productivity in the various production systems is necessary for implementation of improvement strategies.
Lack of government policies that protect indigenous livestock/support its gainful exploitation.	Enabling government policies must be put in place to guide/define the gainful exploitation of indigenous livestock and the roles of other stakeholders (e.g., NGOs).
Lack of infrastructure for routine recording of production and health traits and limited research facilities leading to inadequate performance and pedigree records.	Establishment of national/regional recording and improvement scheme that will attract all stakeholders. Increase research funding and upgrade infrastructure.
Limited information on characterization of national animal genetic resources.	Provide funding to support characterization of production systems, breeds and preservation of local breeds.
Absence of large number of accurately phenotyped animals managed in well-defined contemporary groups with expected breeding values to serve as reference population for genomic selection; limited/lack of genotyping infrastructure; SNP chips derived from different breeds.	Requires a robust national/regional cohesive strategy; more concerted effort required to educate and change the orientation of national policy makers toward funding of research in livestock sector; increased funding by government; private sectors should be encouraged to fund research; put in place infrastructure to deliver genomic services. Develop customized SNP chips based on African livestock populations. Devote financial resources to creating large reference populations with well phenotyped and genotyped animals.
Potential environmental hazards/ethical concerns about genomic approaches to livestock improvement.	Implementation of appropriate biosafety measures and regulatory mechanisms.
Limited expertise or human capacity about genomic breeding approaches for livestock improvement.	Capacity building for all stakeholders (farmers, policy makers, students, and professionals); Train Ph.D. level manpower to measure ‘not too easy to measure’ traits and statistical ability to handle big data. Collaborative research and implementation of improvement techniques with experts in Western countries.
Lack of active and efficient breed associations and no linkages across livestock populations.	Building effective breed association to support producer decisions when needed. Government funding to support establishment of breed associations. Use of artificial insemination even in small holder systems will help to create genetic linkages across livestock populations.
Impatience to implement long-term breeding programs, tendency toward implementation of quick and unstainable breeding methods.	Training and adequate funding to support sustained long term breeding programs. Donor organization to also support sustained long-term programs with participation of producers. Require certain roles of the breeding business section to contribute to sustainability of the development of livestock breeding.
Difficulty of implementing genomic based selection programs.	Appropriate selection programs adapted to each production system implemented; genomic selection suitable for all production systems; selection in nucleus herds using artificial insemination, embryo transfer or embryo sexing; development of appropriate methods/procedures of genotyping and genomic predictions for joint evaluations of small populations.

For farmers/producers to play central roles in the success of any breeding program, they need support from different organizations such as (i) government (to put in place enabling polices, infrastructure, funding, incentives, and markets for their products), (ii) universities and research institutions (to guide, develop up-to-date curriculum, train and provide necessary information for breeding programs, setup and implementation), (iii) international organizations (funding and technological support), (iv) breed societies (maintain records and production characteristics for specific breeds, provide farmers with breed specific information and maintain purebreds). However, the producers themselves need to be actively involved in different breed associations as well as form farmer associations so they can work together to define their priorities (short, medium and long term goals) for implementation in breeding programs. For example; the South African government through its Technology Innovation Agency- TIA initiated a “Beef Genomics Program” in 2014 and a similar program for Dairy was started in 2016 with the goal of expanding to other species in the future (Burrow et al., 2017). Under this scheme, breed associations were expected to develop their own strategy with respect to use of genomic information. This type of approach can be replicated throughout Africa and most developing countries. Unfortunately, there is currently lack of leading roles by most African governments on issues related to livestock development.

The preferences of smallholder farmers is governed by their contextual household characteristics, institutional, and socioeconomic factors (Wale and Yalew, 2007) so their involvement in designing breeding programs is a must. In fact, community based breeding program (CBBP), which refers to improving livestock genetics with the incorporation of farmer participation in selection and breeding activities, has been successfully implemented for several breeds in different countries (Mueller et al., 2015). The CBBP place the farmer’s views, needs and decisions as the most important values and encourage them to participate through the life-cycle of the program from the interception to implementation. The CBBP also allow optimized use of genetic resources and genomic data to support breeding programs suited to specific regions (Kahi et al., 2005; Muniz et al., 2016).

Data collection and storage pose great challenges for African smallholders and even for commercial producers due to the nature of the farming systems (Table 3). At country levels, national improvement schemes to help farmers register and collect data on herd’s performance is scarce. A national milk recording scheme is operational in South Africa and Kenya. In Kenya, however, the willingness of farmers to register with the milk recording scheme and collect data on the productivity of their animals is low. The infrastructure for sample storage is also important for genetic materials. For example, DNA and biological samples need special procedures and materials for collection. The necessary infrastructure to carry out genetic improvement operations is severely constrained in Africa in general. Moreover, lack of baseline epidemiological data on the dynamics and impact of infectious cattle diseases in east Africa seriously limits animal improvement decisions (de Clare Bronsvoort et al., 2013). It is evident that the basic prerequisites for carrying out sample collection in livestock disease outbreaks is lacking for most African countries. It is worthy of note that the current animal health research focus on specific major infectious diseases, particularly tick-borne and tsetse-borne diseases, does not adequately address animal health issues because livestock in the continent are routinely exposed to a wide variety of pathogens. Therefore, the ability to determine correct pathogen effect is important for disease control and quality of data collection.

Most countries have recognized the importance of livestock breeding policy for direction of priorities and activities to be conducted in livestock breeding (Zonabend et al., 2013). However, questions regarding efficiency of implementation of policies and the frequency with which policies are updated to adapt to frequent changes in livestock breeding situations abound. Governments are not only required to draft policies but also to make sure that they are properly implemented. Governments are also required to create access to markets. However, many market problems exist for African countries such as lack of marketing facilities, inadequate marketing organization and methods, and inadequate government policies and marketing-facilitating services.

There is a chronic lack of skilled animal breeders in the African continent which limits the roles of research institutions and universities in designing breeding programs. Universities with Animal Breeding and Genetics programs need to update their curricula to reflect the current state of knowledge in animal breeding and genetics. Students need to be trained in statistics and on how to handle big data associated with advances in the application of knowledge of biotechnology to identify the best animals and make those the parents of the next generation. Also, lack of funding and promotion of research are limitations of African continent based researchers. Moreover, pressure to realize short-term benefits/outcome from research projects impacts negatively sustained gain that can accrue from effective long-term breeding programs. For certain traits, the breeding program needs a long time to realize gains or the impact is slowly accumulated through the years and it is hard to visualize, therefore the need for appropriate methods for measuring the success of breeding programs are required.

Non-governmental organizations (NGOs) are important stakeholders that contribute consultation services, support grass root livestock development programs and are vital partners in tailoring/implementing sustainable breeding programs. NGOs like Heifer Project International^9^, Vétérinaires sans Frontìeres Germany^10^, Send a Cow^11^, etc., have been supporting livestock development projects in the continent. However, greater cooperation between NGOs, international research organizations, national research organizations, universities and farmers will facilitate livestock development programs and widespread adoption of genomic breeding on the continent of Africa.

## Conclusion

The African continent is home to diverse populations of livestock breeds that possess extremely valuable genetic materials but which are not utilized effectively to support economic development or to meet up with increasing demand. Owing to the rich genetic resources and availability of advanced breeding technologies, genomic breeding can be used to speedup livestock development on the continent of Africa. However, the promise and usefulness of genomic tools (especially genomic selection), which have supported livestock gains in many Western countries are yet to be implemented in most of Africa; the major constraints being lack of supportive government policies, funding, nutrition/health challenges, infrastructure and human knowhow. Thus, national governments need to recognize the contribution of livestock production to economic development and the wellbeing of citizens, and put in place enabling policies, necessary infrastructure and funding. Farmers must organize while universities and research institutions should tailor training to the needs of students and farmers. Furthermore, to design effective and sustainable livestock development programs, current production state of breeds and production systems must be adequately characterized through carefully designed investigations for production, reproduction, robustness and fitness traits, and all stakeholders must work together to achieve common goals. The notable success of the community based breeding program could be extended with the inclusion of genomic data as well as by better integration of other stakeholders and clearer government policies. Great opportunities for livestock development exist but all stakeholders must work together to leverage genetic resources for improvement of livestock breeding in Africa.

## Author Contributions

EI-A conceptualized the review, followed by equal distribution of the different sections by EI-A, SP, MB, MA, and DD.

## Conflict of Interest Statement

The authors declare that the research was conducted in the absence of any commercial or financial relationships that could be construed as a potential conflict of interest.

## Footnotes

1 http://www.fao.org/3/MW154en/mw154en.pdf

2 www.faang.org

3 www.ilri.org

4 http://www.nafis.go.ke/livestock/livestock-registration/milk-recording/

5 https://faraafrica.org/

6 https://dgroups.org/fara-net

7 www.illumina.com/

8 www.affymetrix.com/

9 https://www.heifer.org/

10 http://www.vsfg.org/

11 https://www.sendacow.org/
